# A comparison of founder-only and all-pedigree-members genotype-expression association by regression analysis

**DOI:** 10.1186/1753-6561-1-s1-s8

**Published:** 2007-12-18

**Authors:** Young Ju Suh, Hye-Soon Lee, Franak Batliwalla, Wentian Li

**Affiliations:** 1 BK21 Research Division of Medicine and Department of Preventive Medicine, School of Medicine, Ewha Womans University, Seoul 158-710, Korea; 2 The Robert S. Boas Center for Genomics and Human Genetics, Feinstein Institute for Medical Research, North Shore LIJ Health System, 350 Community Drive, Manhasset, New York 11030 USA; 3 Division of Rheumatology, Department of Internal Medicine, Hanyang University Medical College, Seoul 133-792, Korea

## Abstract

Genotype-expression association analysis using linear regression may produce different test results depending on whether founders only or all pedigreed members are used. This difference is not due to the correlation of samples within a pedigree, because linear mixed models have been applied to account for that correlation. We investigated the possibility that the difference is due to a dependence of expression levels on, among other things, the generation number in the pedigree. Indeed, of the 30 or so studied expression quantitative traits, several of them show significant dependence on the generation number. We propose to use all pedigree members in genotype-expression association analyses whenever the complete genotyping information is available.

## Background

The genotype-expression association analyses were performed by Morley et al. [1] and Cheung et al. [2] in unrelated CEPH founders only to avoid the issue of correlated samples. On the other hand, the problem of correlated samples has been dealt with using the mixed model approach in many fields [3], including animal and plant genetics [4,5]. The mixed model with random effects accounts for the correlation by assuming a particular form of covariance structure [6]. The parameters in the mixed model are estimated by the maximum likelihood (or restricted maximum likelihood) approach with iterative algorithms. The correlation among pedigree members apparently can be handled by the mixed model. In fact, a recent paper used the genotype-expression data in Morley et al. [1] as an example to illustrate their version of linear mixed model [7].

In linear regression models (*y ~ ax+b*), the *x *variable in different samples is allowed to be correlated as long as the *y *variable is independent conditional on *x*. Using pedigree members might be a potential problem in genotype-expression association by regression analysis if expression levels in some pedigrees are systematically higher or lower than other pedigrees, thus the parameter *b*, for example, can vary from one pedigree to another in a random fashion. Note that this is a different issue from the correlated samples for allele frequency estimation in case-control analysis [8-10]. In allele frequency estimation, correlation among pedigree members increases the variance of the estimation, while keeping the estimator unbiased [10].

Morley et al. found 27 genes that exhibit strong *cis-*acting genotype-expression (or SNP-eQT) association signal [2]. These signals are believed to be true positives not only because *cis *action makes much biological sense, but also because these genes are under linkage peaks. We used these genes in our study, but relaxed the *cis*-acting requirement: the SNP that shows the strongest association signal will be checked for our comparative study between using founders only and using all pedigree members. This procedure usually selects *trans*-acting SNPs, and thus the chance for false-positive SNP-eQT association is higher.

A comparison of using founders and using all pedigree members might detect something else: consistency between gene expression in founders and in non-founders. If pedigree founders are not a representative subset of samples of a pedigree, using founders only will lead to biased selection. Recent studies have shown that many eQTs may be age-dependent [11], which may provide a biological basis for a possible founder bias. If an eQT is more closely associated with age than with a SNP's genotype, the genotype only partially explains the expression level. We will examine this issue here.

## Methods

### Selection of the SNP-eQT pairs

We selected 28 eQTs: 26 of the 27 gene expressions (there is no probe set in GAW data that matches ICAP-1A) listed in Table 1 of Cheung et al. [2], which show both *cis *linkage signal and strong association signals, and 2 eQTs that are of great interests for our investigation of rheumatoid arthritis, HLA-DRB1 [12] and PTPN22 [13]. After using a filtering procedure, we selected the SNP (out of 2263) that has the strongest association signal with the given eQT by the linear regression of eQT over three genotypes (coded as 0, 1, 2) using the 56 founder samples. Three eQTs (CSTB, DDX17, and HLA-DRB1) contribute two SNP-eQT pairs either because of the second SNP strongly associated with the eQT, or because of the multiple probe sets corresponding to the same eQT, leading to total 31 SNP-eQT pairs. Note that given an eQT, our procedure usually selects the SNP not located in the same region as the gene of that eQT (*tran*-acting); and SNP-eQT pairs used here are mostly different from those in Cheung et al. [2], even though the list of eQTs is the same. For the last subsection of Results section, we also examined all possible SNP-eQT pairs, for 2263 SNPs and 3554 eQTs.

**Table 1:** Averaged percentile value of 28 eQTs

eQT^a^	mean(P)^b^	sd(P)^c^	median(P)^d^
LRAP	55.4	30.61	50
AA827892	52.57	31.16	50
PSPHL	51.63	29.51	50
CPN × 101	62.14	31.05	67.86
CSTB	67.22	29.79	71.43
RPS26	63.18	30.68	71.43
GSTM2	51.28	30.09	50
HLA-DRB2	50.1	29.14	50
IRF5	53.35	30.85	50
HSD17B12	46.74	29.64	42.86
GSTM1	52.27	32.44	46.43
PPAT	59.16	27.14	57.14
DDX17	67.63	26.24	71.43
CTSH	59.7	29.84	62.91
POMZP3	56.65	31.54	57.14
CGI-96	75.87	20.58	78.57
CHI3L2	57.42	30.25	57.14
VAMP8	45.34	30.89	42.86
×10IF3S8	60	28.59	64.29
TM7SF3	64.07	29.1	66.76
IL16	44.2	26.2	42.86
TC × 10A1	54.61	30.17	50
S100A13	43.97	26.06	42.86
SMARCB1	45.37	30.15	35.71
CTBP1	51.41	32.13	50
ZNF85	34.72	24.54	28.57
PTPN22	31.67	19.13	28.57
HLA-DRB1	36.2	28.94	23.08

^a^eQT, name of expression quantitative trait.

^b^mean(P), averaged percentile value of founders for a given eQT.

^c^sd(P), standard deviation of founders' percentile values.

^d^median(P), dmedian of founders' percentile value.

### Linear regression with mixed effects and linear regression of covariates

When all pedigree members are used (194 samples), we considered two models to account for possible pedigree-specific effects on eQT: a random effect on the intercept a (MM1 for mixed model 1):*y*_*ij *_= *a *+ *ε*_*i *_+ *bx*_*ij *_+ *ε*_*ij*_, where *i *is the pedigree index and *j *is the person index; and random effects on both *a *and the slope *b *(MM2 for mixed model 2): *y*_*ij *_+ *a *+ *ε*_*i *_+ (*b *+ *δ*_*i*_)*x*_*ij *_+ *ε*_*ij*_. As for the age effect, we simplified the issue by examining the effect of generation number (1, 2, 3) on expression level, disregarding pedigrees (labeled as "GC model" for generation as a covariate): *y*_*ij *_= *a *+ *bx*_*ij *_+ *c*_2_*g*_2,*ij *_+ *c*_3_*g*_3,*ij *_+ *ε*_*ij*_. Note that the generation covariate is coded as a factor with two dummy variables, g_2 _(1 for generation 2; 0 otherwise) and g_3 _(1 for generation 3; 0 otherwise).

### *t*-Test assuming dominant or recessive model

To check the robustness of linear regression in which an additive model is assumed, we also carried out two *t*-tests for the expression levels by either combining genotypes 0 and 1 as one group (and genotype 2 as the second group), or combining genotype 1 and 2 as a group (and genotype 0 as another group). The best *p*-value of the two tests was selected.

### Averaged percentile of founders

To examine whether founders tend to have higher or lower expression levels with respect to the non-founders, we determined the percentile value for each founder in a pedigree, for a specific eQT. For that eQT, this percentile value was averaged over all pedigrees. If the averaged founder percentile was close to 50%, there was no founder selection bias; and if it was close to 100% or 0%, founders were considered as a biased subset.

### Programs used

Both SAS and R statistical packages were used.

## Results & discussion

### Using founders only and using all pedigree members may lead to different association results

As seen in Additional file 1, a significant association for a SNP-eQT pair using founders only may lose its significance when all pedigree members are used (e.g., the DDX17-rs243404 pair). This observation is somewhat surprising, because there are several reasons to believe the opposite. First, extra samples in the 194-sample data set are offspring of those in the 56-sample data set, so if there is relatedness in their eQTs, it will only reinforce the association signal. Second, with a larger sample size in the 194-sample data set, we would expect a smaller *p*-value, instead of a larger, insignificant one. For the 31 SNP-eQT pairs listed in Additional file 1, 25 pairs' *p*-values are increased in the all-pedigree-member data set (using the naive approach, assuming all samples are independent), despite a tripling of the sample size.

### Checking robustness of result by *t*-tests using dominant and recessive models

Because the linear regression used here implies an additive model, we also carry out two *t*-tests by grouping samples with the heterozygous genotype to those with one of the homozygous genotypes. For founders, the only SNP-eQT pair with *p*-value smaller than 4.4 × 10^-6^(0.01/2263) is CSTB-rs157334 (*p*-value = 1.4 × 10^-6^). When all pedigree members are used, CSTB-rs157334 is still the only pair that is significant at this level (*p*-value = 1.6 × 10^-10^).

### Correcting for sample relatedness by random mixed models

When the MM1 mixed model is applied to the 194-sample data, only two SNP-eQT pairs remain significant at the 4.4 × 10^-6 ^level: CSTB-rs157334 and HSD17B12-rs1334334. When MM2 mixed model is applied, only CSTB-rs157334 exhibits a *p*-value close to that level (5.3 × 10^-6^). Interestingly, this is the only *cis*-acting pair among those listed in Additional file 1. If an eQT has stronger dependence on a SNP genotype and a lower variation from pedigree to pedigree, we expect the significant SNP-eQT association to survive the application of a mixed model, at least with the random effect on the intercept. However, some SNP-eQT pairs lose the test significance in both MM1 and MM2. The MM2 model describes a situation in which not only the eQT varies among pedigrees, but also its degree of dependence on SNP genotype changes with pedigrees. MM2 is a less realistic model than MM1.

### Age/generation effect on eQT

To investigate why all-pedigree-member data set may exhibit a different regression result from the founders-only data set, we examined whether eQT changes from generation to generation. This is a simplified version of examining the age effect, as generation 1 (founders) are older than the second and the third generations. Under the GC regression model described in the Methods section, three *p*-values are listed in Additional file 1: these are for testing zero coefficients for the genotype (*b*), generation 2 (*c*_2_), and generation 3 (*c*_3_). Note that in this regression, pedigree member information is discarded.

Five SNP-eQT pairs in Additional file 1 show significant genotype association at the 4.4 × 10^-6 ^level after accounting for the generation effect: the two pairs mentioned above as well as RPS26-rs720428, CTSH-rs1021639, and GSTM1-rs1039337. On the other hand, significant association with the generation variable has been observed for these eQTs: DDX17 (4.8 × 10^-15^) on generation 2; PTPN22 (2.8 × 10^-20^), CGI-96 (8.4 × 10^-15^), HLA-DRB1 (1.7 × 10^-8^), ZNF85 (3.5 × 10^-8^), IL16 (2.2 × 10^-6^), and CSTB (6.6 × 10^-6^) on generation 3. Interestingly, even though CSTB has a significant association with generation 3, it has an even stronger association with the genotype of rs157334.

A very simple check on whether founders tend to have different expression levels from non-founders is to calculate their percentile value with respect to other samples in the pedigree. For example, for a 14-member pedigree, each member has a percentile value ranging from 1/14 to 1, in an increment of 1/14, and is determined by their expression level of a particular eQT. For 28 eQTs (the probe sets that represent the "consensus sequence" of DDX17 and HLA-DRB1 are selected, whereas those that are "example sequences" are discarded), their average, standard deviation, and median percentile values are listed in Table 1. It is clear that for some eQTs, founders are indeed a biased subset of the pedigree. Most of the examples mentioned above for having a significant generation dependence is regression model also show up in Additional file 1 for having higher or lower averaged founder percentiles: DDX17 (68%), PTPN22 (32%), CGI-96 (76%), HLA-DRB1 (36%), ZNF85 (35%), and CSTB (67%).

**Figure 1:**
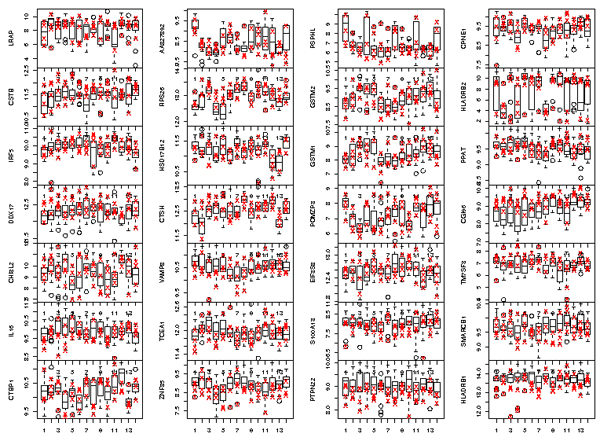
**Box-and-whisker plot of 28 eQTs for 14 pedigrees**. The founders' expression level is marked by crosses (four founders per pedigree).

To view more directly the relative location of founders' expression with respect to other pedigree members, Figure 1 shows the box-and-whisker plot of the 28 eQTs, with the founders marked by crosses.

Yet another simple check for the founder effect on expression is the two-way ANOVA with pedigree as one factor and founder/non-founder as the second factor. Our result shows that 1) pedigree-dependence of the expression is significant for most eQTs (with the exception of four to five eQTs); 2) founder-dependence of expression is significant for nine eQTs at the *p*-value = 0.001 level. These nine eQTs are CSTB, RPS26, DDX17, CGI96, TM7SF3, IL16, ZNF85, PTPN22, and HLA-DRB1, consistent with the result from the percentile value calculation; 3) for the founder-pedigree interaction term, four eQTs are significant at the *p*-value = 0.001 level.

### One-stage versus two-stage analysis

Although it does not apply to the GAW data, in a practical setting, genome-wide genotyping information may only be available for the first stage of a study. In this situation, one may select SNPs that show promising association signals, and only type these SNPs for the rest of the pedigree (second stage) in order to save cost. It is also suggested by Van Steen et al. that two-stage design also helps to ease the multiple testing problem [14]. If the whole genome genotyping information is available for all pedigree members, one should use all samples in the analysis, while correcting the sample correlation by appropriate procedures (such as the mixed model procedure discussed here).

To test where a two-stage design would lead us, we imagine a hypothetical situation in which we do not have the genotyping information for non-founders. Then we would first exhaustively perform all possible genotype-expression linear regression analyses for the 56 founders. There are more than 8 millions possible SNP-eQT pairs, leading to an equal number of *p*-values. From these results, for each eQT, the minimum *p*-values of all 2263 SNPs can be recorded. There are 47 eQTs that have a minimum *p*-value lower than 4.4 × 10^-6^. Now we assume in the second-stage that these selective SNPs are typed for non-founders. For the corresponding eQT-SNP pair, another linear regression analysis on all 194 members can be carried out, as well as a linear regression analysis with the generation variable as covariates. If we do that, among these 47 eQT-SNP pairs, only two remain significant at the 4.4 × 10^-6 ^level for the 194-sample data set: CSTB-rs157334 and HSD17B12-rs1334334. Interestingly, these two pairs are the only overlap between the 47-pair set and the 31-pair set listed in Additional file 1. Furthermore, the two pairs are also the only ones showing significant association with genotype after the generation effect has been removed.

Because the eQTs listed in Table 1 of Cheung et al. [2] (and Additional file 1 here) are selected based on a larger data set and, more importantly, extra information (e.g., linkage signal), they stand at a better chance to be true positives. The fact that the only two eQTs whose significant association with a SNP survive the test using all pedigree members cautions us on the practice of not using all available data, but only relying on a subset of the data set.

## Conclusion

We have shown that besides pedigree founders, pedigree members can also be used in a SNP-eQT association analysis. The issue of relatedness among pedigree members can be handled by mixed models, either with one or two random effects to account for pedigree-specific expression variations. Using all pedigree members has other advantages, such as increasing the sample size, checking the consistency among samples, and detecting possible age dependence in expression levels.

## List of Abbreviations

CEPH: Centre d'Etude du Polymorphisme Human

GAW: Genetic Analysis Workshop 15

GC: generation as a covariate (linear regression model)

eQT: expression quantitative trait

MM: mixed model (with 1 or 2 random effects)

SNP: single-nucleotide polymorphism

## Competing interests

The author(s) declare that they have no competing interests.

## Supplementary Material

Additional file 1 Comparison of *p*-values in regression analyses for 31 selected SNP-eQT pairs Click here for file
