# Supplementary material for: A comparison of founder-only and all-pedigree-members genotype-expression association by regression analysis
Source: BMC Proc. 2007 Dec 18;1(Suppl 1):S8. doi: 10.1186/1753-6561-1-s1-s8 (PMC2367500; doi:10.1186/1753-6561-1-s1-s8)
Supplement: Additional file 1 — Comparison of p-values in regression analyses for 31 selected SNP-eQT pairs [file 1753-6561-1-S1-S8-S1.doc]

## Comparison of *p*-values in regression analyses for 31 selected SNP-eQT pairs

| **eQT^a^** | **chE^b^** | **SNP^c^** | **chS^d^** | **p(56)^e^** | **p(194)^e^** | **p(MM1)^f^** | **p(MM2)^f^** | **p(GC,b)^g^** | **p(c2)^g^** | **p(c3)^g^** |
| --- | --- | --- | --- | --- | --- | --- | --- | --- | --- | --- |
| LRAP | 5q | rs753639 | 18 | 4.2×10^-5^ | 0.00044 | 0.053 | 0.053 | 0.00045 | 0.62 | 0.28 |
| AA827892 | 20q | rs1507031 | 16 | 0.00048 | 0.81 | 0.25 | 0.26 | 0.64 | 0.98 | 0.024 |
| PSPHL | 7p | rs966365 | 1 | 0.00085 | 7.8×10^-5^ | 0.0031 | 0.027 | 8.0×10^-5^ | 0.39 | 0.52 |
| CPN×101 | 20q | rs1382461 | 13 | 0.00034 | 0.19 | 0.021 | 0.044 | 0.16 | 0.0082 | 0.12 |
| CSTB | 21q | rs1573334 | 21 | 3.3×10^-8^ | 8.3×10^-14^ | 7.9×10^-13^ | 5.3×10^-6^ | 4.0×10^-14^ | 0.0082 | 6.6×10^-6^ |
| CSTB | 21q | rs1999811 | 1 | 4.5×10^-6^ | 0.043 | 0.0072 | 0.0072 | 0.042 | 0.017 | 2.6×10^-5^ |
| RPS26 | 12q | rs720428 | 4 | 0.00057 | 1.9×10^-7^ | 0.11 | 0.11 | 8.3×10^-8^ | 0.76 | 0.0023 |
| GSTM2 | 1p | rs1458132 | 8 | 0.00011 | 0.0031 | 0.066 | 0.066 | 0.0024 | 0.078 | 0.11 |
| HLA-DRB2 | 6p | rs1395579 | 16 | 9.4×10^-6^ | 8.4×10^-5^ | 0.042 | 0.12 | 8.6×10^-5^ | 0.78 | 0.59 |
| IRF5 | 7q | rs317588 | 3 | 0.00079 | 0.22 | 0.063 | 0.077 | 0.23 | 0.85 | 0.94 |
| HSD17B12 | 11p | rs1334334 | 1 | 1.8×10^-6^ | 3.2×10^-11^ | 1.9×10^-6^ | 0.00045 | 4.4×10^-11^ | 0.60 | 0.031 |
| GSTM1 | 1p | rs1039337 | 9 | 0.00018 | 3.7×10^-7^ | 0.013 | 0.034 | 3.0×10^-7^ | 0.48 | 0.23 |
| PPAT | 4q | rs914671 | 10 | 0.0020 | 0.75 | 0.89 | 0.89 | 0.74 | 0.0059 | 0.24 |
| DDX17 | 22q | rs1886204 | 13 | 0.00031 | 0.047 | 0.024 | 0.10 | 0.047 | 0.57 | 0.15 |
| DDX17 | 22q | rs243404 | 19 | 7.4×10^-6^ | 0.012 | 0.036 | 0.036 | 0.00029 | 4.8×10-^15^ | .00016 |
| CTSH | 15q | rs1021639 | 1 | 2.8×10^-5^ | 1.0×10^-8^ | 0.0058 | 0.0058 | 1.0×10^-8^ | 0.21 | 0.16 |
| POMZP3 | 7q | rs930548 | 1 | 2.8×10^-5^ | 0.30 | 0.073 | 0.24 | 0.31 | 0.81 | 0.59 |
| CGI-96 | 22q | rs2055708 | 11 | 0.00086 | 0.98 | 0.56 | 0.45 | 0.97 | 0.85 | 8.4×10^-15^ |
| CHI3L2 | 1p | Rs889121 | 19 | 0.00084 | 0.022 | 0.11 | 0.12 | 0.016 | 0.51 | 0.16 |
| VAMP8 | 2p | rs1462411 | 5 | 0.00047 | 0.0037 | 0.0072 | 0.022 | 0.0026 | 0.059 | 0.96 |
| ×10IF3S8 | 16p | rs1562180 | 8 | 8.4×10^-5^ | 0.0037 | 0.00078 | 0.00078 | 0.0026 | 0.023 | 0.0063 |
| TM7SF3 | 12p | rs1022590 | 14 | 0.00096 | 0.00032 | 0.0017 | 0.17 | 0.00014 | 0.62 | .00045 |
| IL16 | 15q | rs948215 | 11 | 0.00040 | 0.0035 | 0.20 | 0.21 | 0.00075 | 0.81 | 2.2×10^-6^ |
| TC×10A1 | 8q | rs1037973 | 16 | 0.00020 | 0.012 | 0.26 | 0.26 | 0.011 | 0.51 | 0.68 |
| S100A13 | 1q | rs729685 | 4 | 0.0015 | 0.12 | 0.0079 | 0.012 | 0.11 | 0.029 | .00012 |
| SMARCB1 | 22q | rs890440 | 8 | 0.00063 | 0.12 | 0.072 | 0.066 | 0.18 | 0.49 | 0.0021 |
| CTBP1 | 4p | rs1079638 | 16 | 9.0×10^-5^ | 0.020 | 0.026 | 0.028 | 0.027 | 0.15 | 0.17 |
| ZNF85 | 19p | rs1936875 | 10 | 8.7×10^-5^ | 0.0031 | 0.048 | 0.039 | 0.0013 | 0.34 | 3.5×10^-8^ |
| PTPN22 | 1p | rs984068 | 9 | 0.00042 | 0.52 | 0.84 | 0.84 | 0.26 | 0.18 | 2.8×10^-20^ |
| HLA-DRB1 | 6p | rs941838 | 14 | 5.9×10^-5^ | 0.30 | 0.55 | 0.55 | 0.17 | 0.0022 | 0.020 |
| HLA-DRB1 | 6p | rs941838 | 14 | 0.00014 | 0.85 | 0.82 | 0.82 | 0.37 | 8.6×10^-6^ | 1.7×10^-8^ |

## ^a^eQT, name of the gene which is represented by an expression (HLA-DRB1 is represented by two expression QTs: 209312_x_at and 215193_x_at).

## ^b^chE, the chromosome arm the eQT is located.

## ^c^SNP, the marker that shows the strongest association with the given eQT.

## ^d^chS, chromosome on which the SNP is located.

## ^e^p(56), p(194), *p*-value for linear regression of eQT on SNP genotype (0, 1, 2) using 56 (194) pedigree founders (pedigree members).

## ^f^p(MM1), p(MM2), *p*-value for mixed model with one (two) random effect using 194 pedigree members.

## ^g^p(GC, b), p(c2), p(c3), *p*-values, of the linear regression with generation as a covariate, for testing zero-coefficient for the SNP genotype, generation 2, and generation 3.
